# Bidirectionally Regulating Gamma Oscillations in Wilson-Cowan Model by Self-Feedback Loops: A Computational Study

**DOI:** 10.3389/fnsys.2022.723237

**Published:** 2022-02-21

**Authors:** XiuPing Li, ZhengHong Li, WanMei Yang, Zhen Wu, JunSong Wang

**Affiliations:** ^1^School of Biomedical Engineering, Tianjin Medical University, Tianjin, China; ^2^Department of Psychology, Tianjin University of Technology and Education, Tianjin, China; ^3^College of Big Data and Internet, Shenzhen Technology University, Shenzhen, China

**Keywords:** Wilson-Cowan model, gamma oscillations, self-feedback loops, bifurcation analysis, spectrum analysis

## Abstract

The Wilson-Cowan model can emulate gamma oscillations, and thus is extensively used to research the generation of gamma oscillations closely related to cognitive functions. Previous studies have revealed that excitatory and inhibitory inputs to the model can modulate its gamma oscillations. Inhibitory and excitatory self-feedback loops are important structural features of the model, however, its functional role in the regulation of gamma oscillations in the model is still unclear. In the present study, bifurcation analysis and spectrum analysis are employed to elucidate the regulating mechanism of gamma oscillations underlined by the inhibitory and excitatory self-feedback loops, especially how the two self-feedback loops cooperate to generate the gamma oscillations and regulate the oscillation frequency. The present results reveal that, on one hand, the inhibitory self-feedback loop is not conducive to the generation of gamma oscillations, and increased inhibitory self-feedback strength facilitates the enhancement of the oscillation frequency. On the other hand, the excitatory self-feedback loop promotes the generation of gamma oscillations, and increased excitatory self-feedback strength leads to the decrease of oscillation frequency. Finally, theoretical analysis is conducted to provide explain on how the two self-feedback loops play a crucial role in the generation and regulation of neural oscillations in the model. To sum up, Inhibitory and excitatory self-feedback loops play a complementary role in generating and regulating the gamma oscillation in Wilson-Cowan model, and cooperate to bidirectionally regulate the gamma-oscillation frequency in a more flexible manner. These results might provide testable hypotheses for future experimental research.

## Introduction

Gamma oscillation is a rhythmic electrical activity ranged from 30 to 80 Hz (Buzsáki, 2009; Wang et al., 2011), which is widely present in the thalamus, cortex and hippocampus of animal and human brains (Womelsdorf and Fries, 2007), and is closely related to the cognitive function of animals and humans (Gevins et al., 1997; Engel et al., 2001; Bartos et al., 2007; Cabral et al., 2014). On the other hand, the abnormal gamma oscillation is an important cause of cognitive impairment and neurological disorders such as schizophrenia (Lewis et al., 2005; Light et al., 2006), autism (Orekhova et al., 2007; Milne et al., 2009) and language learning disorder (Heim et al., 2011). Therefore, studying the regulating mechanism of gamma oscillations is of great significance for understanding brain cognitive function and cognitive impairment.

So far, important progress has been made in physiological experimental research on gamma oscillations. *In vitro* experimental studies have shown that inhibitory interneurons are the key factor in the generation of gamma oscillations, and inhibitory postsynaptic potential (IPSP) is a necessary condition for the synchronization of gamma oscillations (Colgin and Moser, 2010). The synaptic connection strength between neurons plays an important role in the generation of gamma oscillations. Belluscio et al. (2012) showed that the interaction between excitation and inhibitory neurons can promote the generation of gamma oscillations. Atallah and Scanziani (2009) studied the potential regulating mechanism of gamma oscillations by recording the membrane potential inside and outside of rat cells, indicating that the connection strength between excitatory and inhibitory neurons can quickly adjust the frequency of gamma oscillations. Ray and Maunsell (2011) recorded local field potential (LPF) in the V1 brain area of rhesus monkeys to detect the origin of oscillations in different gamma bands. Ray et al. (2013) studied the effect of excitation and inhibition balance on gamma oscillations by recording the local field potential (LPF) in the MT area of rhesus monkeys, and found that visual stimuli would change the balance of excitability and inhibitory activity, and then adjust the gamma oscillations. Recent experimental studies have shown that visual stimuli plays an important role in inducing gamma oscillations and frequency modulation (Chen et al., 2017, 2020; Saleem et al., 2017; Veit et al., 2017; Welle and Contreras, 2017). Veit et al. (2017) used optogenetic technology to conduct mouse experiments and found that SOM (somatostatin) cells were stimulated to induce gamma oscillations, and inhibitory SOM cells would decrease the energy of gamma oscillations.

Computational research is another important method to understand the regulating mechanism of gamma oscillations. Geisler et al. (2005) found that the input of the neuron model has a substantial impact on gamma oscillations, and the excitatory and inhibitory synaptic time constants can predict the model’s frequency. The Wilson-Cowan model is a mesoscopic firing rate model proposed by Wilson and Cowan (1972), describing the interaction between excitatory and inhibitory populations. The model can simulate rich neural dynamics (Wilson and Cowan, 1972; Destexhe and Sejnowski, 2009), thus is widely used in research on gamma oscillation regulating mechanisms (Srinivasan et al., 2013; Jadi and Sejnowski, 2014b; Veltz and Sejnowski, 2015; Keeley et al., 2019). Srinivasan et al. (2013) showed that the background input has an important effect on the response of Wilson-Cowan model, and reproduced the important experimental phenomenon that theta oscillation modulates the gamma oscillation through simulation. Veltz and Sejnowski (2015) studied the regulation of gamma rhythm by periodic input and the phase-amplitude coupling mechanism between two neural groups by constructing two interconnected Wilson-Cowan models. Jadi and Sejnowski (2014b) found that the super-linear response of the sigmoid functions in the inhibitory population of the Wilson-Cowan model play a key role in modulating the frequency and power of the simulated cortical gamma oscillations, furthermore, the balance of excitatory and inhibitory inputs into the model determine the frequency and power of oscillations. Jadi and Sejnowski (2014a) used two different stimulation methods to regulate the gamma oscillations in the excitatory and inhibitory coupled neural network, and revealed that stimulating the inhibitory neuron group can enhance the gamma oscillation power while stimulating the excitatory neuron group makes the gamma oscillation frequency larger. Keeley et al. (2019) have found that the time constants of excitatory and inhibitory populations have an important influence on the gamma oscillations in an extended Wilson-Cowan model. To sum up, the above-mentioned research on the regulating mechanism of gamma oscillations in the Wilson-Cowan model mainly focuses on the effect of excitatory and inhibitory inputs, sigmoid functions, and time constants of excitatory and inhibitory populations on gamma oscillations.

A self-feedback loop is a ubiquitous structure of neural circuits and neural networks. Computational studies have found that it has an important regulatory effect on brain electrical activity (Wilson and Cowan, 1972, 1973; Youssofzadeh et al., 2015). Moran et al. (2007, 2008) found that the Jansen-Rit neural mass model (Jansen and Rit, 1995) can successfully simulate gamma oscillations by introducing an inhibitory self-feedback loop into the model. Ursino et al. (2010) revealed that extended Jansen-Rit neural mass model (Wendling et al., 2010) can simulate gamma and beta oscillations simultaneously by introducing an inhibitory self-feedback loop into the fast inhibitory interneuron population. Wang et al. (2020) studied the effect of inhibitory self-feedback on the dynamics and oscillations of the subthalamic nucleus (STN) and globus pallidus (GPe) neural circuit model, and demonstrated that inhibitory self-feedback exert an important effect on the generation and regulation of beta oscillations. These previous studies have shown that inhibitory self-feedback has an important effect on the dynamic characteristics and oscillation behavior of the Jansen-Rit neural mass model and its extended versions.

Excitatory and inhibitory self-feedback loops are important structure features of the Wilson-Cowan model, however, it still remains to be addressed how the inhibitory self-feedback loop exerts an effect on the regulation of gamma oscillations in the model, furthermore, it is still unknown whether or not the excitatory self-feedback loop also plays a role in the regulation mechanism, especially the way the two loops cooperate to regulate the gamma oscillations. Here, by conducting bifurcation analysis and spectrum analysis, we aim to elucidate regulating mechanism of gamma oscillations in the Wilson-Cowan model underlined by the two self-feedback loops.

The rest of this paper is organized as follows. In the section “Model and Methods,” the Wilson-Cowan model is described, which can produce gamma oscillations within certain parameter regions. In the section “Results,” by combining bifurcation analysis and spectrum analysis, we explore how inhibitory and excitatory self-feedback loops regulate gamma oscillations, respectively, and especially focus on elucidating the synergistic regulating mechanism underlined by the interaction of the two self-feedback loops. Finally, the conclusions are given in the section “Discussions.”

## Model And Methods

### Model

**FIGURE 1:**
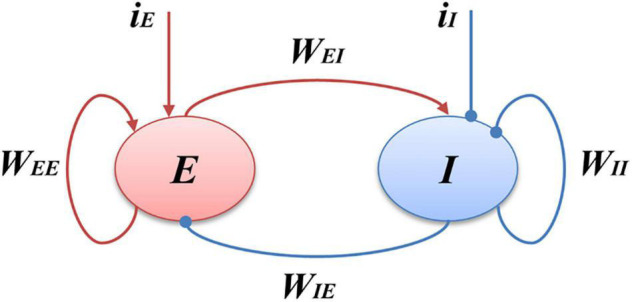
Schematic of the Wilson-Cowan model. *E* represents the excitatory populations, *I* stands for the inhibitory populations, the red arrows indicate the excitatory projections and blue arrows show the inhibitory projections, *i*_*I*_ is the external inputs of the *I* populations, and *i*_*E*_ is the external inputs of the *E* populations.

The Wilson-Cowan model is a firing rate model at the mesoscopic level proposed by Wilson and Cowan (1972). The schematic diagram of the model is shown in Figure 1, where “*E*” represents excitatory population, “*I*” inhibitory population, *i*_*I*_ and *i*_*E*_ stands for inhibitory and excitatory inputs to the model, *W*_*EI*_ and *W*_*IE*_ represent the coupling strength between excitatory and inhibitory populations, and *W*_*EE*_ and *W*_*II*_ the excitatory and inhibitory self-feedback connection strength, respectively. This model is mainly composed of excitatory and inhibitory populations with self-feedback loops, and can emulates the interaction between the excitatory and inhibitory populations.

The mathematical formulation of Wilson-Cowan model is formulated as follows:


(1) $$\tau_{I}\,\frac{\text{d}\,r_{I}}{d\,t}=-r_{I}+G_{I}\,\left(W_{I\,E}\,{\text{r}}_{E}-W_{I\,I}\,r_{I}+i_{I}\right)$$

(2) $$\tau_{E}\,\frac{\text{d}\,r_{E}}{d\,t}=-r_{E}+G_{E}\,\left(W_{E\,E}\,{\text{r}}_{E}-W_{E\,I}\,r_{I}+i_{E}\right)$$

Where *r*E** and *r*I** are the outputs of the model, representing the firing rate of excitatory and inhibitory populations, respectively.

*G*_*E*_(⋅) and *G_*I*_(⋅)* are two response functions describing the non-linear dynamical properties of the model, respectively, and the two response functions are given by:


(3) $$G_{E/I}\,\left(x\right)=\frac{1}{1+{\text{e}}^{-m_{E/I}\,\left(x-\theta_{E/I}\right)}}-\frac{1}{1+e^{m_{E/I}•\theta_{E/I}}}$$

The parameter values in the model are given in Table 1 (Wilson and Cowan, 1972; Jadi and Sejnowski, 2014b). In this work, we mainly explore the role of the excitatory and inhibitory self-feedback strength *W*_*EE*_ and *W*_*II*_ played in the regulation of gamma oscillations, other parameters remain the default values as Table 1. The parameters were chosen where the generating oscillation frequency of the model was in the gamma band (Ledoux and Brunel, 2011; Jadi and Sejnowski, 2014b).

**TABLE 1:** Parameters interpretation and values in the Wilson-Cowan model.

Parameters (units)	Interpretation	Values
i_*E*_	External input to excitatory population	2
i_*I*_	External input to inhibitory population	7
*W*_*IE*_	Strength of connections between *I-E*	20
*W*_*EI*_	Strength of connections between *E–I*	26
τ_*E*_ /ms	Average synaptic time constant for excitatory population	20
τ_*I*_ /ms	Average synaptic time constant for inhibitory population	10
m_*E*_, m_*I*_, θ_*E*_, θ_*I*_	Parameters of non-linear S function	m_*E*_ = 1;m_*I*_ = 1, θ_*E*_ = 5;θ_*I*_ = 20
*W*_*EE*_	Strength of self-excitatory feedback	16
*W*_*II*_	Strength of self-inhibitory feedback	1

### Methods

Neural electrical activity in the brain has rich non-linear characteristics, and the bifurcation analysis method is an effective means to understand the non-linear dynamic behavior of the brain (Gallez and Babloyantz, 1991; Grimbert and Faugeras, 2006; Spiegler et al., 2010; Touboul et al., 2011; Xiaofei and Junsong, 2014; Dong and Zhu, 2020). When one or several parameters of the dynamic system are varied, the behavior of the system undergoes a qualitative change, such as the transformation between stable and unstable state, the appearance and disappearance of limit cycle, the emergence of chaos, and so on. These sudden changes are called bifurcations. The Wilson-Cowan model is a typical non-linear system with rich and complex dynamics (Borisyuk and Kirillov, 1992; Ledoux and Brunel, 2011), for example, a stable equilibrium point in the model corresponds to the resting state of neural mass, and the appearance of Hopf bifurcation points in the model indicates the transformation of the system from a stable equilibrium point to limit cycle oscillation which often corresponds to the periodic oscillatory electrical activity of the neural mass. Therefore, the model parameters highly influence the non-linear dynamic behavior of the Wilson-Cowan model, which plays an important role in revealing the principle of how gamma oscillation is generated and regulated.

XPPAUT (Ermentrout, 2002) and MatCont (Dhooge et al., 2003) are commonly used software packages for bifurcation analysis to draw the dynamic trajectory diagram of the system as model parameters change. In this study, XPPAUT software is used for one-parameter bifurcation analysis, and Matcont is used for two-parameter bifurcation analysis. Particularly, only the positive values in the bifurcation diagram are biologically relevant, and the negative values are purely served as a mathematical description in order to generate the full bifurcation curves.

**FIGURE 2:**
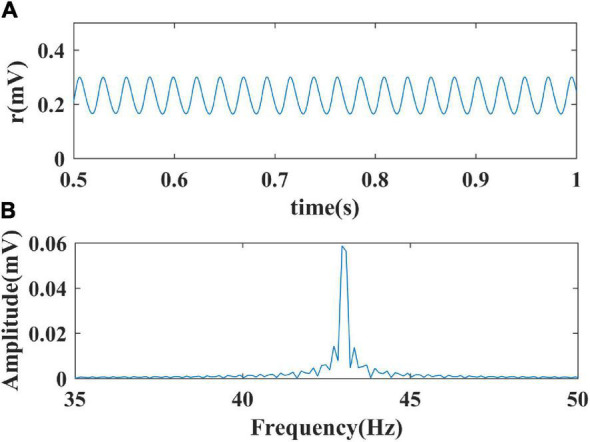
The process of spectral analysis. **(A)** The simulated output signal of the model; **(B)** The spectral analysis result of the output.

Through giving the fixed parameters and external inputs, the signal output of the model with time changing was simulated. Next, the Fourier transform was performed to determine the oscillation frequency of the model’s output signals. Finally, the frequency distribution of the simulated limit cycle oscillations in a two-dimensional model parameter plane was obtained. The spectral analysis process is shown in Figure 2. In the present study, we conduct the Fourier transform of the signal to plot frequency curves with respect to model parameters and frequency distribution diagrams in a two-dimensional parameter plane by using MATLAB software. The Wilson-Cowan model was integrated with the Runge-Kutta numerical integration method with a time step of 0.001 s to solve the differential equations at a zero initial condition (Ashwin et al., 2015).

## Results

In this section, we conduct a one-parameter bifurcation to determine the ranges of inhibitory and excitatory self-feedback strength generating limit cycle oscillations, thus probing how inhibitory and excitatory self-feedback loops generate gamma oscillations in the Wilson-Cowan model. Furthermore, through two-parameter bifurcation analysis, we explored how inhibitory and excitatory self-feedback loops interact with other model parameters to exert influence on the gamma oscillations, especially how the two loops cooperate to regulate the gamma oscillations. In addition, to probe how the two loops regulate oscillation frequency, we perform spectrum analysis to obtain the distribution diagram of oscillation frequency.

### Regulating Gamma Oscillations by Inhibitory Self-Feedback Loop

#### One-Parameter Bifurcation Analysis With Respect to Inhibitory Self-Feedback Strength

**FIGURE 3:**
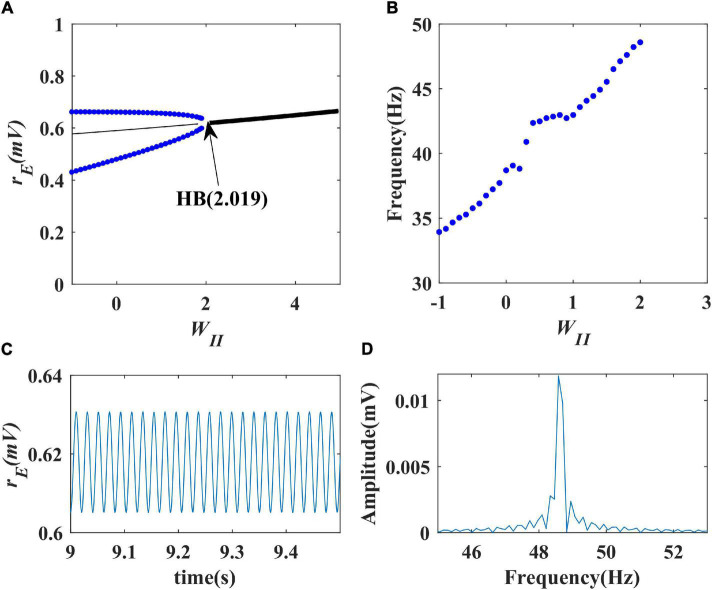
The impact of inhibitory self-feedback strength *W*_*II*_ on the dynamical behavior of the Wilson-Cowan model. **(A)** The one-parameter bifurcation diagram with respect to *W*_*II*_, The Hopf bifurcation point is labeled as “HB,” respectively; **(B)** Frequency curve versus *W*_*II*_; **(C)** Simulation result (*W*_*II*_ = 2); **(D)** Spectrum analysis result (*W*_*II*_ = 2).

Firstly, we performed a one-parameter bifurcation analysis with respect to the inhibitory self-feedback strength. The bifurcation diagram is shown in Figure 3A, where HB represents the Hopf bifurcation point (*W_*II*_* = 2.019), the black solid line the stable equilibrium point, and the black dashed line the unstable equilibrium point. The model produces a limit cycle oscillation at the range of *W_*II*_* ≤ 2.019, corresponding to the blue curve area in Figure 3A. As shown in Figure 3A, the model exhibits a limit cycle oscillating state when the value of inhibitory self-feedback strength is small; with the increase of the inhibitory self-feedback strength, at the Hopf bifurcation point, the model switches from limit cycle oscillation to a unique stable fixed point state, and the oscillations disappear. Thus the bifurcation result indicates that increased inhibitory self-feedback strength is not conducive to the generation of gamma oscillations.

Next, to explore how the inhibitory self-feedback loop regulates the oscillation frequency, we determined the oscillation frequency curve *versus* inhibitory self-feedback strength, shown in Figure 3B. It is suggested that increased inhibitory self-feedback strength results in the oscillation frequency to increasing.

Furthermore, we conducted simulations to verify the bifurcation analysis result and oscillation frequency curve were correct. The simulation and spectrum analysis results are shown in Figures 3C,D, respectively, which demonstrates that the bifurcation analysis result and oscillation frequency curve are correct.

#### Two-Parameter Bifurcation Analysis With Respect to Inhibitory Self-Feedback Strength and Inhibitory Input or Coupling Strength

**FIGURE 4:**
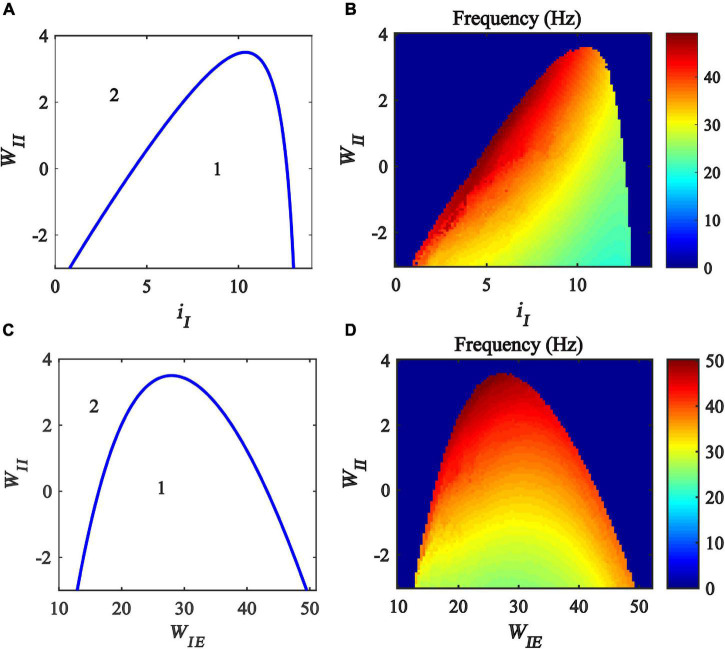
Influences of the interaction of inhibitory self-feedback and inhibitory input or coupling strength on the dynamic behavior of the Wilson-Cowan model. **(A)** The codimension two bifurcations with respect to *i*_*I*_ and *W*_*II*_; **(C)** The codimension two bifurcations with respect to *W*_*IE*_ and *W*_*II*_; **(B,D)** Oscillation frequency distribution diagrams. The area labeled 1 represents limit cycle oscillations, the area labeled 2 represents fixed-point state, and the blue curve represents Hopf bifurcation curves.

Previous studies have found that excitatory and inhibitory inputs can modulate the gamma oscillations in the Wilson-Cowan model (Jadi and Sejnowski, 2014a,b; Gu et al., 2020). To explore how the inhibitory self-feedback loop interacts with inhibitory input to regulate gamma oscillations, We perform a two-parameter bifurcation analysis with respect to inhibitory self-feedback strength *W*_*II*_ and the inhibitory input *i*_*I*_. The two-parameter bifurcation diagrams were obtained, as shown in Figure 4A, where the blue curve is the Hopf bifurcation curve. The behaviors of the model are divided into two regions in the plane (*i_*I*_,W_*II*_*), denoted as 1 and 2 with different dynamic properties. The region denoted “*1*” exhibits a limit cycle oscillation state, and the other region denoted “*2*” corresponds to stable fixed point behavior. The bifurcation results indicate that, with the increase of the inhibitory self-feedback strength, the regions of inhibitory input to generate limit cycle oscillation decrease, suggesting that the inhibitory self-feedback loop suppresses the generation of limit cycle oscillation.

To probe how the inhibitory self-feedback loop interacts with inhibitory input to regulate the oscillation frequency, the oscillation frequency distribution diagram in the plane (*i_*I*_, W_*II*_*) is determined as shown in Figure 4B, indicating that the oscillation frequency increases with the increase of the inhibitory self-feedback strength.

Moreover, some studies have claimed that connecting strength between excitatory and inhibitory neurons has an effect on modulating cortical gamma oscillations (Tiesinga and Sejnowski, 2009; Li et al., 2011; Buzsáki and Wang, 2012). Inspired by this idea, we carried out further two-parameter bifurcation analysis and spectrum analysis with respect to inhibitory self-feedback *W*_*II*_ and coupling strength *W*_*IE*_ of the Wilson-Cowan model. The bifurcation result and oscillation frequency distribution diagram are illustrated in Figures 4C,D, respectively. Obviously, increased inhibitory self-feedback strength prevents the model from generating gamma oscillations, and leads to the increase of the oscillation frequency, the same conclusions as that obtained according to Figures 4A,C.

#### Impact of the Inhibitory Self-Feedback Loop on Two-Parameter Bifurcation Results With Respect to Inhibitory and Excitatory Inputs

Previous studies have revealed that inhibitory and excitatory inputs play an important role in the generation and regulation of gamma oscillations (Jadi and Sejnowski, 2014b). To discover whether or not the inhibitory self-feedback loop exerts an impact on the regulating law underlined by the two inputs, we performed a two-parameter bifurcation analysis with respect to inhibitory and excitatory inputs under different inhibitory self-feedback strengths.

**FIGURE 5:**
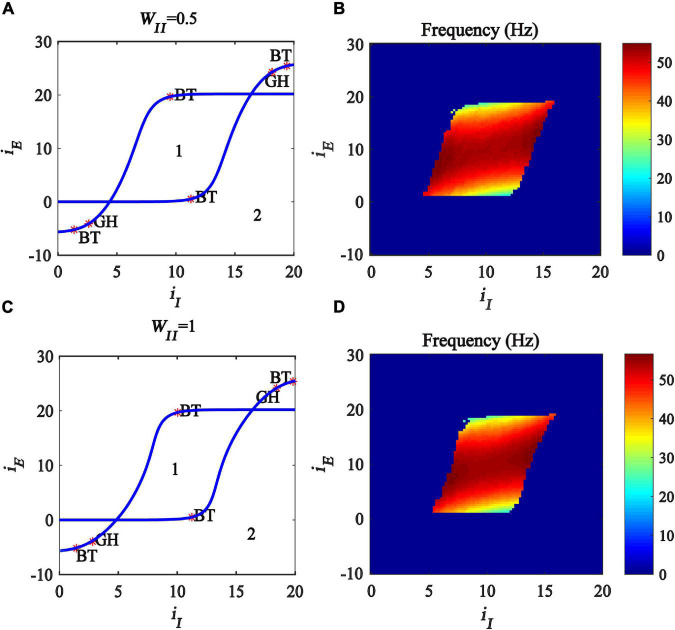
Influences of the interaction of inhibitory and excitatory inputs on the dynamic behavior of the Wilson-Cowan model with different inhibitory self-feedback strength. **(A,C)** Are the two-parameter bifurcations with respect to *i*_*I*_ and *i*_*E*_ under different *W*_*II*_; **(B,D)** Frequency distribution diagrams. **(A,B)** *W_*II*_* = 0.5. **(C,D)** *W*_*II*_ = 1. The blue curve represents Hopf bifurcation curves, GH stands for the generalized Hopf bifurcation, BT represents Bogdanov-Takens bifurcation, the area labeled 1 represents limit cycle oscillations.

The bifurcation results are shown in Figures 5A,C, respectively, where BT (Bogdanov-Takens Bifurcation) is the intersection point of saddle node bifurcation and Hopf bifurcation, GH (Generalized Hopf Bifurcation) the intersection point of subcritical and supercritical Hopf bifurcation, the blue curves represent the Hopf bifurcation, and the region denoted “*1*” represents the limit cycle oscillation state. The oscillation frequency distribution diagrams are plotted, shown in Figures 5B,D, and the oscillation frequency ranges from 30 to 55 Hz, corresponding to the gamma band oscillation. The results demonstrate that, with the increase of the inhibitory self-feedback strength, the region generating gamma oscillations in the plane (*i_*I*_, i_*E*_*) becomes small, which means that inhibitory self-feedback is not conducive to the generation of gamma oscillations.

### Regulation of Gamma Oscillations by Excitatory Self-Feedback Loop

Besides the inhibitory self-feedback loop, the excitatory self-feedback loop is the other connection motif in the Wilson-Cowan model. However, the role this loop plays still remains to be addressed. In this section, we conduct bifurcation analysis and spectrum analysis to elucidate the regulation mechanism of gamma oscillations in the Wilson-Cowan model that is underlined by the excitatory self-feedback loop.

#### One-Parameter Bifurcation Analysis With Respect to Excitatory Self-Feedback Strength

**FIGURE 6:**
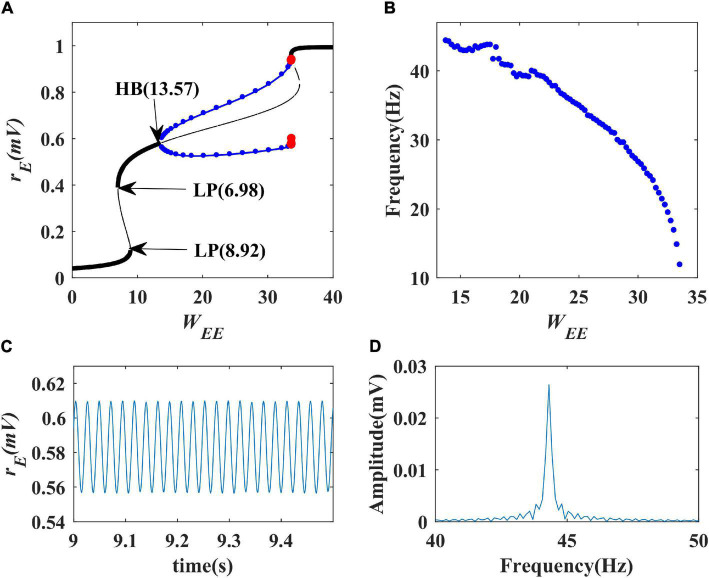
The impact of excitatory self-feedback strength *W*_*EE*_ on the dynamical behavior of the Wilson-Cowan model. **(A)** The one-parameter bifurcation diagram with respect to *W*_*EE*_, The Hopf bifurcation point is labeled as “HB,” LP stands for limit point bifurcation; **(B)** Frequency curve *versus W_*EE*_*; **(C)** Simulation result (*W_*EE*_* = 14); **(D)** Spectrum analysis result (*W*_*EE*_ = 14).

We first performed a one-parameter bifurcation analysis with respect to excitatory self-feedback strength. The bifurcation diagram is shown in Figure 6A where the blue point represents a stable limit cycle oscillation, and the red point an unstable limit cycle oscillation. The model is at a stable state and does not produce oscillation when the excitatory self-feedback strength is smaller than the Hopf bifurcation point (HB point) value *W*_*EE*_ = 13.57; with the increase of the excitatory self-feedback strength, the model exhibits limit cycle oscillation in the region of 13.57<*W*_*EE*_<35, and switches from the limit cycle oscillation to monostable behavior at *W*_*EE*_ = 35. The bifurcation diagram shows that the Wilson-Cowan model produces limit cycle oscillations as the excitatory self-feedback strength increases, and the oscillation disappears when it exceeds a certain value. According to Table 1, the standard value of excitatory self-feedback strength is less than 35, thus in general the excitatory self-feedback loop is conducive to the generation of gamma oscillations.

The oscillation frequency curve versus the excitatory self-feedback strength is shown in Figure 6B, suggesting that the oscillation frequency decreases with the increase of excitatory self-feedback strength. The simulation and spectrum analysis results at the value of *W*_*EE*_ = 14 are illustrated in Figures 6C,D, respectively, indicating the correctness of the analysis results. Thus, we can conclude that excitatory self-feedback plays an important role in regulating the oscillation frequency.

#### Two-Parameter Bifurcation Analysis With Respect to Excitatory Self-Feedback Strength and Inhibitory Input or Coupling Strength

**FIGURE 7:**
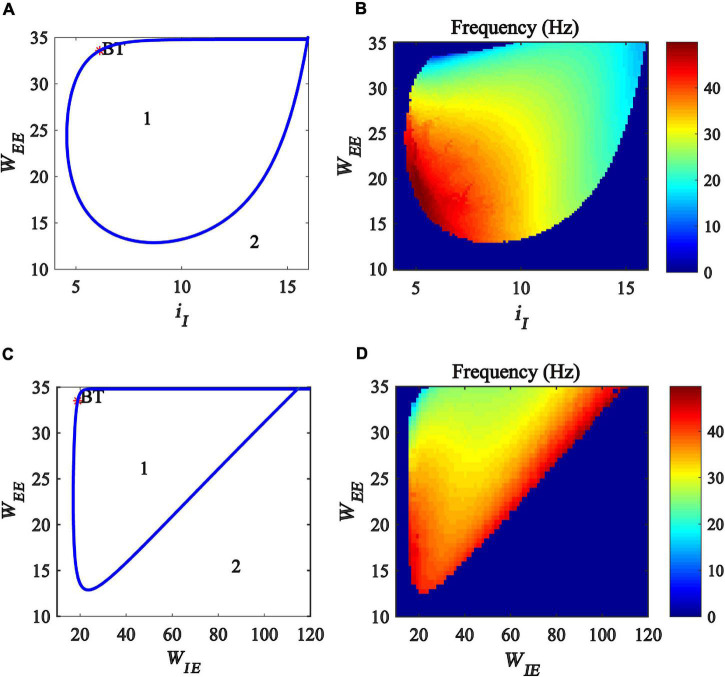
Influences of the interaction of excitatory self-feedback strength and inhibitory input or coupling strength on the dynamic behavior of the Wilson-Cowan model. **(A)** The codimension two bifurcations with respect to *i*_*I*_ and *W*_*EE*_; **(C)** The two-parameter bifurcations with respect to *W*_*IE*_and*W*_*EE*_; **(B,D)** Frequency distribution diagrams. The area labeled 1 represents limit cycle oscillations, the blue curve represents Hopf bifurcation curves, and BT represents Bogdanov-Takens bifurcation.

In this subsection, we explore the regulation of gamma oscillations by the interaction between excitatory self-feedback and inhibitory input or the coupling strength between excitatory and inhibitory populations. To this end, we created a two-parameter bifurcation with respect to (*i_*I*_, W_*EE*_*) and (*W_*IE*_, W_*EE*_*), respectively. The bifurcation diagrams are shown in Figures 7A,C, where the blue curve represents Hopf bifurcation. The region denoted “*1*” indicates that the model is at a limit cycle oscillation state, and the region denoted “*2*” represents a stable fixed point state. The bifurcation diagram shows that, with the increase of excitatory self-feedback strength *W*_*EE*_, the oscillation region in the plane (*i_*I*_, W_*EE*_*) and (*W_*IE*_, W_*EE*_*) become large, indicating that the excitatory self-feedback loop promotes the generation of gamma oscillations.

Furthermore, we investigate the influence of the excitatory self-feedback loop on the oscillation frequency. The frequency distribution diagrams in the plane (*i_*I*_, W_*EE*_*) and (*W_*IE*_, W_*EE*_*) are obtained, as shown in Figures 7B,D. It was suggested that the oscillation frequency decreases with the increase of the excitatory self-feedback strength.

#### Impact of the Excitatory Self-Feedback Loop on Two-Parameter Bifurcation Results With Respect to Inhibitory and Excitatory Inputs

**FIGURE 8:**
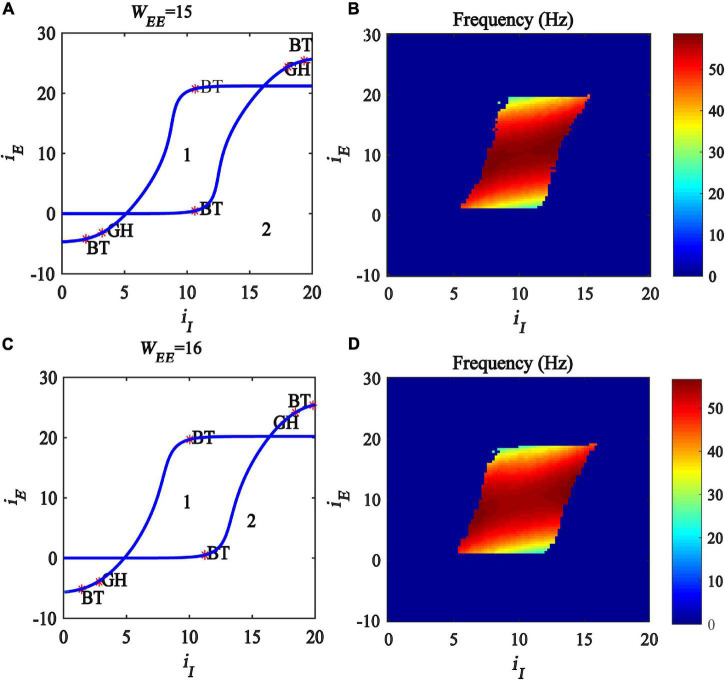
Influences of the interaction of inhibitory and excitatory inputs on the dynamic behavior of the Wilson-Cowan model with different excitatory self-feedback strength. **(A,C)** The codimension two bifurcations with respect to *i*_*I*_ and *i*_*E*_ under different *W*_*EE*_; **(B,D)** Frequency distribution diagrams. **(A,B)** *W_*EE*_* = 15. **(C,D)** *W_*EE*_* = 16. The blue curve is the Hopf bifurcation curve, GH stands for the generalized Hopf bifurcation, BT represents Bogdanov-Takens bifurcation and Area labeled 1 represents limit cycle oscillations.

In this subsection, we further investigate the influence of excitatory self-feedback strength on the regulating mechanism underlined by the excitatory and inhibitory inputs. We conduct a two-parameter bifurcation analysis with respect to (*i_*I*_, i_*E*_*) under different excitatory self-feedback strength *W*_*EE*_, and the bifurcation diagrams are illustrated in Figures 8A,C, respectively. The oscillation frequency distribution diagrams are shown in Figures 8B,D, corresponding to the gamma band oscillation. The results demonstrate that the areas generating limit cycle oscillations in the plane (*i_*I*_,i_*E*_*) become large with increased excitatory self-feedback strength *W*_*EE*_, indicating that the excitatory self-feedback loop is conducive to the generation of gamma oscillations. It is suggested that the excitatory self-feedback loop has an important effect on the gamma oscillation regulating law underlined by inhibitory and excitatory inputs.

### Synergistic Regulation of Gamma Oscillations by Inhibitory and Excitatory Self-Feedback Loops

By combining the results in sections “Regulating Gamma Oscillations by Inhibitory Self-Feedback Loop” and “Regulation of Gamma Oscillations by Excitatory Self-Feedback Loop,” we can conclude that both excitatory and inhibitory self-feedback loops are crucial for the regulation of gamma oscillations under the Wilson-Cowan model. Thus an interesting question is how the two loops interact to exert an influence on the gamma oscillation. To address this problem, we further investigated the synergistic regulation of gamma oscillations by the cooperation of inhibitory and excitatory self-feedback loops in the Wilson-Cowan model.

**FIGURE 9:**
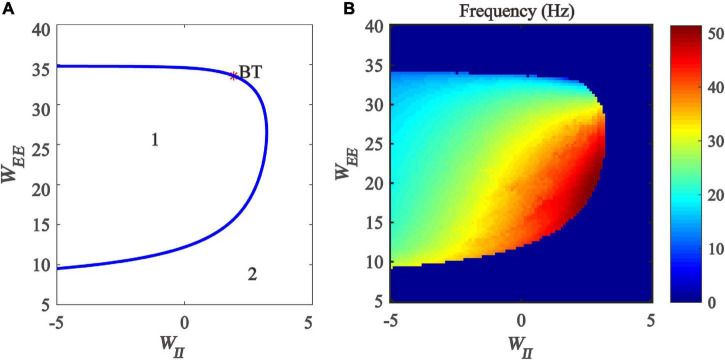
The impact of the interaction between inhibitory self-feedback strength *W*_*II*_ and excitatory self-feedback strength *W*_*EE*_ on the dynamical behavior of the Wilson-Cowan model. **(A)** The two-parameter bifurcations with respect to *W*_*II*_ and *W*_*EE*_; **(B)** Frequency distribution diagrams. The blue curve represents the Hopf bifurcation curves, BT represents Bogdanov-Takens bifurcation, the area labeled 1 represents limit cycle oscillations, and the area labeled 2 represents fixed-point state.

Firstly, we drew the two-parameter bifurcation diagram with respect to inhibitory and excitatory self-feedback strength, as shown in Figure 9A. The blue Hopf bifurcation curve divides the two-dimensional parameter plane (*W_*II*_, W_*EE*_*) into two regions, where the region denoted “*1*” stands for the limit cycle oscillation state, and the region labeled “*2*” is the monostable state. The bifurcation diagram shows that, with the increase of excitation self-feedback strength, the oscillation region of the model is increased, which means that the excitatory self-feedback loop promotes the generation of gamma oscillations. while increased inhibitory self-feedback strength results in the oscillation region of the model becoming small, indicating that the inhibitory self-feedback loop is not conducive to the generation of gamma oscillations.

Next, the oscillation frequency distribution diagram is determined, shown as in Figure 9B. It should be noted that the part of Figure 9B where the model parameters are negative does not have any biological significance, but only serves as a mathematical description of the model. Figure 9B indicates that the frequency of the limit cycle oscillation is mainly gamma band activity, and increased inhibitory self-feedback strength leads to the increase of the oscillation frequency, while increased excitatory self-feedback strength causes the oscillation frequency to decrease.

### Theoretical Analysis

In this subsection, we further conduct a theoretical analysis to provide explanations on the generating and regulating mechanism of oscillations underlined by the excitatory and inhibitory self-feedback loops. Linear control theory is employed to bridge the relationship of self-feedback parameter *W*_*II*_ and *W*_*EE*_ with the oscillation characteristics of the Wilson-Cowan model.

The mathematical descriptions of the inhibitory and excitatory populations of the model, i.e., Eqs. (1, 2), can be reformulated in the form of


(4) $$\left\{ \begin{array}{c} \tau_{I}\,\frac{\text{d}\,r_{I}}{d\,t}=-r_{I}+y_{I}\\ y_{I}=G_{I}\,\left(W_{I\,E}\,{\text{r}}_{E}-W_{I\,I}\,r_{I}+i_{I}\right) \end{array}\right.$$

(5) $$\left\{ \begin{array}{c} \tau_{E}\,\frac{\text{d}\,r_{E}}{d\,t}=-r_{E}+y_{E}\\ y_{E}=G_{E}\,\left(W_{E\,E}\,{\text{r}}_{E}-W_{E\,I}\,r_{I}+i_{E}\right) \end{array}\right.$$

**FIGURE 10:**
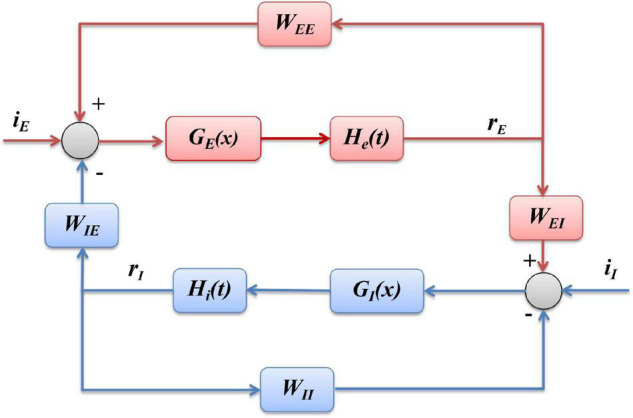
Block diagram of Wilson-Cowan model. The blue part represents the inhibitory population, and the red part is the excitatory population, respectively.

According to the structure shown as Figure 1, and the mathematical formulation as Eqs. (1, 2) of Wilson-Cowan model, we can derive the block diagram of the model, demonstrated as Figure 10, where the inhibitory population (blue part) is mainly composed of linear part *h*_*I*_(*t*) and non-linear sigmoid function *G*_*I*_(*x*), and excitatory population (red part) includes linear *h*_*E*_(*t*) and non-linear *G*_*E*_(*x*), where *x*represents the input of a sigmoid function, *h*_*I*_(*t*)and *h*_*E*_(*t*)are the impulse response functions of $\tau_{I}\,\frac{\text{d}\,r_{I}}{d\,t}=-r_{I}+y_{I}$ and $\tau_{E}\,\frac{\text{d}\,r_{E}}{d\,t}=-r_{E}+y_{E}$, respectively.

Firstly, we will explore how the inhibitory self-feedback loop exerts an effect on the oscillation of the Wilson-Cowan model. We approximate the inhibitory sigmoid function *G*_*I*_(*x*)in the inhibitory population with the first-order Taylor expansion $K_{I}=\frac{d\,G_{I}\,\left(x\right)}{d\,x}$. The Laplace transformation of the linear function *h*_*I*_(*t*)can be formulated as HI⁢(s)=1τ⁢sI+1.

When there is no inhibitory self-feedback loop in the inhibitory population, i.e., *W*_*II*_ = 0, the transfer function of the inhibitory population is defined as the ratio of the Laplace transform of the output variable to the Laplace transform of the input variable, thus we derived it as follows


(6) $$\phi_{I}\,\left(s\right)=K_{I}\,H_{I}\,\left(s\right)=\frac{K_{I}}{\tau_{I}\,s+1}$$

Then, by substituting *s* = *j*ω into Eq. (6), we obtain Fourier transformation, i.e., the frequency response characteristics of the inhibitory population as:


(7) $$\phi_{I}\,\left(j\,\omega \right)=K_{I}\,H_{I}\,\left(j\,\omega \right)=\frac{K_{I}}{j\,\tau_{I}\,\omega +1}$$

Obviously, the equivalent gain and time constant of the inhibitory population without an inhibitory self-feedback loop are *K*_*I*_ and τ_*I*_, respectively.

Furthermore, according to the blue part of the block diagram shown in Figure 10, we can derive


$$R_{I}\,\left(s\right)=\left[I_{I}\,\left(s\right)-R_{I}\,\left(s\right)\,W_{I\,I}\right]\,\phi_{I}\,\left(s\right)$$

which can be formulated as


$$R_{I}\,\left(s\right)\,\left[1+W_{I\,I}\,\phi_{I}\,\left(s\right)\right]=I_{I}\,\left(s\right)\,\phi_{I}\,\left(s\right)$$

Thus, we obtained the closed-loop transfer function of the inhibitory population with inhibitory self-feedback, i.e., the ratio of the Laplace transform of the output variable to the Laplace transform of the input variable of the inhibitory population, as follows:


(8-1) $$\phi_{I}^{\prime }\,\left(s\right)=\frac{R_{I}\,\left(s\right)}{I_{I}\,\left(s\right)}=\frac{\phi_{I}\,\left(s\right)}{1+W_{I\,I}\,\phi_{I}\,\left(s\right)}$$

where *R*_*I*_(*s*) and *I*_*I*_(*s*) are the Laplace transform of the output variable *r*_*I*_(*t*)and the Laplace transform of the input variable *i*_*I*_(*t*) of the inhibitory population, respectively.

Substituting Eq. (6) into Eq. (8–1), we further derived


(8-2) $$\phi_{I}^{\prime }\,\left(s\right)=\frac{K_{I}\cdot H_{I}\,\left(s\right)}{1+W_{I\,I}\,K_{I}\cdot H_{I}\,\left(s\right)}$$

By substituting *s* = *j*ω into Eq. (8–2), we further obtained the frequency response characteristics of the inhibitory population as:


$$\phi_{E}^{\prime }\,\left(j\,\omega \right)=\frac{K_{I}\cdot H_{I}\,\left(j\,\omega \right)}{1+W_{I\,I}\,K_{I}\cdot H_{I}\,\left(j\,\omega \right)}=\frac{K_{I}}{1+W_{I\,I}\,K_{I}+\tau_{I}\,j\,\omega }=\frac{K_{I}/\left(1+W_{I\,I}\,K_{I}\right)}{1+\tau_{I}\,j\,\omega /\tau_{I}\,j\,\omega \,\left(1+W_{I\,I}\,K_{I}\right)}=\frac{K_{I}^{\prime }}{1+\tau_{I}^{\prime }\,j_{\omega }}$$

where


(9) $$\left\{ \begin{array}{c} K_{I}^{\prime }=K_{I}\,/\,\left(1+W_{I\,I}\,K_{I}\right)\\ \tau_{I}^{\prime }=\tau_{I}\,/\,\left(1+W_{I\,I}\,K_{I}\right) \end{array}\right.$$

and $K_{I}^{\prime }$ is the equivalent gain, and $\tau_{I}^{\prime }$ the equivalent time constant of the inhibitory population, respectively.

Thus we can draw the following conclusion


(10) $$\left\{ \begin{array}{c} K_{I}^{\prime }<K_{I}\\ \tau_{I}^{\prime }<\tau_{I} \end{array}\right.$$

Equations 9, 10 demonstrate that the inhibitory self-feedback loop, i.e., the introduction of *W*_*II*_ into the inhibitory population, causes both the equivalent gain and time constant of the inhibitory population to be decreased. It should be pointed that this conclusion does not depend on the selection of the other model parameters. According to the control and dynamics theory (Franklin et al., 1994), we can conclude that the decreased equivalent gain prevents the model from generating oscillation, obviously, the decreased equivalent time constant results in the oscillation frequency increased.

For the excitatory population, using the same analysis method as the above mentioned, we determined the transfer function of the excitatory population without excitatory self-feedback as follows


(11) $$\phi_{E}\,\left(s\right)=\frac{R_{E}\,\left(s\right)}{I_{E}\,\left(s\right)}=K_{E}\,H_{E}\,\left(s\right)=\frac{K_{E}}{\tau_{E}\,s+1}$$

where $K_{I}=\frac{d\,G_{E}\,\left(x\right)}{d\,x}$ is the linear approximation of the excitatory sigmoid function *G*_*E*_(*x*)in the excitatory population with the first-order Taylor expansion, and $H_{e}\,\left(s\right)=\frac{1}{\tau_{E}\,s+1}$ the Laplace transformation of the linear function *h*_*E*_(*t*).

The transfer function of the excitatory population with excitatory self-feedback can be derived as follows


(12-1) $$\phi_{E}^{\prime }\,\left(s\right)=\frac{R_{E}\,\left(s\right)}{I_{E}\,\left(s\right)}=\frac{\phi_{E}\,\left(s\right)}{1+W_{E\,E}\,\phi_{E}\,\left(s\right)}$$

Substituting Eq. (11) into Eq. (12–1), we further derived


(12-2) $$\phi_{E}^{\prime }\,\left(s\right)=\frac{K_{E}\cdot H_{E}\,\left(s\right)}{1+W_{E\,E}\,K_{E}\cdot H_{E}\,\left(s\right)}$$

By substituting *s* = *j*ω into Eq. (12–2), we obtained


$$\phi_{E}^{\prime }\,\left(j\,\omega \right)=\frac{K_{E}\,/\,\left(1-W_{E\,E}\,K_{E}\right)}{1+\tau_{E}\,j\,\omega \,/\,\left(1-W_{E\,E}\,K_{E}\right)}=\frac{K_{E}^{\prime }}{1+\tau_{E}^{\prime }\,j\,\omega }$$

where


(13) $$\left\{ \begin{array}{c} K_{E}^{\prime }=K_{E}\,/\,\left(1-W_{E\,E}\,K_{E}\right)\\ \tau_{E}^{\prime }=\tau_{E}\,/\,\left(1-W_{E\,E}\,K_{E}\right) \end{array}\right.$$

and $K_{E}^{\prime }$ is the equivalent gains, and $\tau_{E}^{\prime }$ the equivalent time constants of the excitatory population without and with excitatory self-feedback loop, respectively.

Obviously, we can draw the following conclusion for the excitatory population


(14) $$\left\{ \begin{array}{c} K_{E}^{\prime }>K_{E}\\ \tau_{E}^{\prime }>\tau_{E} \end{array}\right.$$

Thus, the excitatory self-feedback loop results in the increased gain and time constants of the excitatory population, which demonstrates that the excitatory self-feedback loop facilities the generation of oscillation, and causes the oscillation frequency to decrease.

## Discussion

From the view point of biological meaning, the self-feedback loop corresponds to an autapse (Auto-synapse) in a neural circuit. Separate from synapses between different neurons, an autapse is an unusual kind of synapse that connects to the neuron itself, which was first discovered in 1972 in the pyramidal cell of neocortex cerebri by Van der Loos and Glaser (1972). Experimental studies have found that autapses has been observed in various brain regions such as in the neocortex, hippocampus, cerebellum, and striatum (Loos and Glaser, 1972; Bekkers and Stevens, 1991; Gábor et al., 1997; Bacci et al., 2003). Especially, autapses can form a self-feedback loop in a neuron. Meanwhile, the autapse plays a rich role in the regulation of neuron firing and neural rhythm. Biological experiments suggest that inhibitory self-feedback can suppress neuron firing (Saa Da et al., 2009), while excitatory self-feedback can promote neuron firing (Yin et al., 2018). In addition, Connelly has found that self-feedback plays a crucial role in enhancing the synchrony of the membrane potential across the entire network during the neocortical gamma oscillations (Connelly, 2014). Recently, by using the axon patch-clamp recording technique, research has shown that a self-feedback loop in the neuron can promote neuronal responsiveness, burst firing, and coincidence detection in these neocortical principal cells (Yin et al., 2018). Different from these previous studies, the present results reveal the regulating mechanism of neural oscillations underlined by excitatory and inhibitory autapses at the level of population. Furthermore, we conducted a theoretical analysis to provide explanation of these regulating mechanisms, which can shed light on a deeper understanding of the role of excitatory and inhibitory autapses play in achieving brain functions and causing neural disorders.

Gamma oscillations are closely related to brain functions, for example, learning and memory. Most previous studies focused on the synapse between different neurons how to influence brain functions; the investigations in this paper might provide an avenue to understand autapse underlined brain function mechanisms. Moreover, abnormal neural oscillations are an important biomarker of many kinds of neural diseases, such as epilepsy, Parkinson’s disease, etc. The present results can bridge the relationship between the neural oscillations and excitatory and inhibitory autapses, which may provide some guidelines and predictions for future experimental studies on how autapses exert an impact on brain functions and neural diseases.

## Conclusion

The Wilson-Cowan model can generate gamma oscillations and is extensively employed to study the regulation mechanism of gamma oscillations. Excitatory and inhibitory self-feedback loops are typical connection modes of the model, however, it is unclear how the two self-feedback loops exert an effect on the regulation of gamma oscillations in the model. The results suggest that inhibitory self-feedback is not conducive to the generation of gamma oscillations, while excitatory self-feedback promotes it. Moreover, The increased inhibitory self-feedback strength causes the oscillation frequency to increase, and with the increase of excitatory self-feedback strength, the oscillation frequency is decreased. It should be noted that these conclusions do not depend on the selection of the other model parameters. Taken together, these results reveal that inhibition and excitatory self-feedback loops play a complementary role in the generation and regulation of gamma oscillations. Through the cooperation of the two self-feedback loops, a flexible bidirectional regulation of gamma oscillations in the Wilson-Cowan model can be achieved.

In the present study, there are some limitations as follows: The study builds on the parameters from the previous research, such as the works conducted by Ledoux & Brunel and Jadi & Sejnowski. As opposed to the previous study of Jadi and Sejnowski, the present study mainly focuses on exploring how the inhibitory and excitatory self-feedback loops regulate the neural oscillations of the Wilson-Cowan model and has no direct relationship to external inputs corresponding to sensory inputs. The results have revealed that the excitatory and inhibitory self-feedback loops have an important effect on the underlined regulatory mechanisms of the model inputs, neural oscillations, which implies that the two self-feedback loops may exert impact on the response of the model to some sensory inputs. In the future, experimental research could be further conducted to verify the present conclusions.

## Data Availability Statement

The original contributions presented in the study are included in the article/supplementary material, further inquiries can be directed to the corresponding author.

## Author Contributions

XL completed the result analysis and wrote the first draft of the manuscript. ZL and WY participated in manuscript design and results analysis. ZW and JW contributed to conception and design of the study. JW was responsible for result analysis, manuscript writing, and revision. All authors contributed to manuscript revision, read, and approved the submitted version.

## Conflict of Interest

The authors declare that the research was conducted in the absence of any commercial or financial relationships that could be construed as a potential conflict of interest.

## Publisher’s Note

All claims expressed in this article are solely those of the authors and do not necessarily represent those of their affiliated organizations, or those of the publisher, the editors and the reviewers. Any product that may be evaluated in this article, or claim that may be made by its manufacturer, is not guaranteed or endorsed by the publisher.
